# Effect of cultivation mode on the production of docosahexaenoic acid by *Tisochrysis lutea*

**DOI:** 10.1186/s13568-018-0580-9

**Published:** 2018-03-30

**Authors:** Hao Hu, Lin-Lin Ma, Xiao-Fei Shen, Jia-Yun Li, Hou-Feng Wang, Raymond Jianxiong Zeng

**Affiliations:** 1 CAS Key Laboratory of Urban Pollutant Conversion, Department of Chemistry, University of Science and Technology of China, Hefei, 230026 People’s Republic of China; 2 Anhui Water Conservancy College, Hefei, 231603 People’s Republic of China; 3 Advanced Laboratory for Environmental Research and Technology, USTC-CityU, Suzhou, 215123 People’s Republic of China; 4 The First Affiliated Hospital of Anhui University of Traditional Chinese Medicine, Hefei, 230031 People’s Republic of China

**Keywords:** Docosahexaenoic acid (DHA), Total fatty acids (TFAs), Mixotrophy, Heterotrophy, *Tisochrysis lutea*

## Abstract

In this study, *Tisochrysis lutea* was cultivated in mixotrophic and heterotrophic cultures with glycerol as a carbon source and with glucose and acetate for comparison; autotrophic cultivation was the control group without a carbon source. It was found that *T. lutea* used glycerol and did not use glucose and acetate under mixotrophy. Mixotrophy slightly elevated the docosahexaenoic acid (DHA) and total fatty acids (TFA) content in the dry-weight and enhanced the DHA and TFA production in medium (41.3 and 31.9% respectively) at the end of a 16-day cultivation, while heterotrophy reduced the DHA content and TFA production. Under the mixotrophy, the glycerol contribution to the DHA production (16.19 mg/L) and the TFA production (97.8 mg/L) was not very high and the DHA yield [2.63% chemical oxygen demand (COD)] and TFA yield (13.1% COD) were also very low. Furthermore, *T. lutea* using glycerol had a period of adaptation, indicating that *T. lutea* was not an ideal microalga for organic carbon utilization.

**Electronic supplementary material:**

The online version of this article (10.1186/s13568-018-0580-9) contains supplementary material, which is available to authorized users.

## Introduction

Docosahexaenoic acid (DHA) has a high medical and nutritional value; it promotes visual acuity and neural development (Boelen et al. 2013), and reduces the risk of some diseases such as cardiovascular, arthritis, diabetes, and obesity related breast cancer (Biscione et al. 2007; Lee et al. 2012; Manni et al. 2017). As a result, DHA has received worldwide attention in past decades. The primary commercial source of DHA is marine fish oil (Khozin-Goldberg et al. 2011); however, DHA has certain disadvantages, such as unstable quality, a fishy odor, environmental pollution, a long production period, high processing costs, and easy oxidation (Maehre et al. 2015). Microalgae might be the most promising alternative because they are the primary producers of DHA and the DHA in fish oil is derived from microalgae (Ryckebosch et al. 2014). In addition, microalgae-derived DHA overcomes the disadvantages of DHA from marine fish (Martins et al. 2013).

Many microalgae species contain DHA (Martins et al. 2013). *Tisochrysis lutea* has been recognized as one of the most suitable species for DHA production due to its fast growth (Alkhamis and Qin 2016) and high DHA content [12–14% in total fatty acids (TFAs)] (Tzovenis et al. 1997, 2003). The DHA content of *T. lutea* is much higher than other microalgae such as *Phaeodactylum tricornutum* (0.98% TFAs) (Qiao et al. 2016), *Isochrysis galbana* (6.84% TFAs) (Liu et al. 2013), *Pyramimonas* sp. (5.8% TFAs), and *Pavlova lutheri* (9.6% TFAs) (Guiheneuf and Stengel 2013). Researchers have also made many efforts to enhance DHA content by various means in the past years. Sukenik and Wahnon (1991) used a high light intensity and nitrogen concentration to improve the DHA content of *T. lutea* to 17.6 mol% of TFAs. *T. lutea* was cultured by Saoudihelis et al. (1994) in a chemostat and produced a high DHA content (16 mg/g) at high growth rates. The effect of the N:P ratio on the DHA content of *T. lutea* was studied by Rasdi and Qin (2015) and the results indicated that N:P = 20:1 enhanced the DHA content to 8.1% of TFAs.

However, most of the studies reported in the literature were based on traditional autotrophic cultivation. Some culture conditions affect growth of *T. lutea* under autotrophy, such as temperature (Marchetti et al. 2012), salinity (O’Shea et al. 2010), illumination (del Pilar Sanchez-Saavedra et al. 2016). Among them the light condition is an important factor for growth of *T. lutea*. The low microalgal growth rates and biomass productivity in autotrophic culture are due to self-shading and light limitations (Lowrey et al. 2016), which inevitably results in low DHA production. Mixotrophic or heterotrophic culture is a promising approach to promote biomass productivity and secondary metabolite production in some microalgal species such as *Spirulina platensis* (Andrade and Costa 2007), *Chlorella vulgaris, Haematococcus* sp.*, Nannochloris* sp.*, and Scenedesmus* sp. (Andruleviciute et al. 2014). Research on the heterotrophic and mixotrophic culture of *T. lutea* has just begun. *T. lutea* was grown in a mixotrophic culture as reported by Alkhamis and Qin (2016), but the DHA production was low (13.94 mg/L) and moreover, a heterotrophic culture was not investigated.

For a mixotrophic or heterotrophic culture, the organic carbon source plays a crucial role to provide energy and maintain growth in the dark or under limited light conditions (Wang et al. 2014). Different microalgae species can use different carbon sources in mixotrophic or heterotrophic growth condition. *Spimlina platensis* (Chen and Zhang 1997), *Nannochloropsis* sp. (Xu et al. 2004), and *C. vulgaris* (Shen et al. 2015a) use glucose as a carbon source. *Scenedesmus obliquus* (Shen et al. 2015b) and *P. tricornutum* (Wang et al. 2012) use acetate, and *Neochloris oleabundans*, *Botryococcus Braunii*, and *Dunaliella* sp. use glycerol (Choi and Lee 2015). Additionally, some microalgae use several carbon sources such as glucose, acetate, and glycerol (Sahin et al. 2018; Wang et al. 2012; Zhang et al. 2013). There are few research studies on the utilization of carbon sources by *T. lutea*. Alkhamis and Qin (2016) reported that *T. lutea* used glycerol in a mixotrophic culture but it was not reported whether other carbon sources (glucose, acetate, etc.) were utilized and whether heterotrophic growth occurred.

In this study, we selected *T. lutea* and investigated three organic carbon sources (glucose, acetate, and glycerol) for biomass growth and DHA production. The impact on the DHA and TFA production by changing the cultivation mode from autotrophy to mixotrophy or heterotrophy was also examined. The nutrient assimilation and the DHA and TFA production were measured, the productivity and yield [% chemical oxygen demand (COD)] of the DHA and TFAs were calculated, and the fatty acid (FA) composition was analyzed. The results will improve understanding of *T. lutea* for DHA production under different cultivation modes.

## Materials and methods

### Strain and pre-culture condition

*Tisochrysis lutea* CCMP1324 was obtained from the National Center for Marine Algae and Microbiota (NCMA), East Boothbay, USA. The culture medium was f/2 medium (Guillard 1975) with Na_2_SiO_3_ removed (f/2-Si medium). The stock solutions of NaNO_3_, NaH_2_PO_4_•2H_2_O, and the microelements of the metal salts from the f/2 medium were prepared and stored at 4 °C, added to the artificial seawater from f/2 medium, and then autoclaved at 121 °C for 25 min before use. The vitamin solution from f/2 medium was prepared and stored at 4 °C and was filtered through a 0.22-μm filter (Merck Millipore, USA) for sterilization before being added to the medium. *T. lutea* was pre-cultivated in a 1-L Erlenmeyer flask with 800 mL of the medium and with magnetic stirrer in the bottom keeping 150–200 rpm. Six 40-W straight fluorescent tubes (Phillips) were placed 20 cm above the flasks in a dark room. The illumination intensity was 3000 lx with a photoperiodicity of 14:10 (light for 14 h and dark for 10 h). The dark room maintained 23 ± 2 °C by air-conditioner. The initial medium pH was adjusted by 1.2 M HCl or 1 M NaOH to 6.5 ± 0.3 before autoclaving and was slightly increased to 7.5 ± 0.5 after autoclaving, then pH of culture medium was measured and adjusted to 7.0–8.0 every 4 day. The pH was determined by the pH detector (PHSJ-3F, INESA scientific instrument Co. Ltd, Shanghai, China). After 12-day cultivation, the medium was ready to be inoculated.

### Experimental setup

There were two experiments in this study, organic carbon sources experiment (experimental set-up 1) and cultivation mode experiment (experimental set-up 2). In experimental setup 1, glucose, glycerol, and sodium acetate (47,829, 1,295,607, 791,741, respectively, Sigma, USA) as carbon sources with 5 g/L each were added to the f/2-Si medium and no organic carbon source was added as a control group, and each group had three parallel setups. *T. lutea* was cultivated in a 500-mL Erlenmeyer flask with 300 mL of the medium and 30 mL of the pre-culture microalgal suspension. The light conditions were the same as in the pre-culture conditions. After 10-day cultivation, the biomass, nitrogen, phosphorus, and organic substrate concentrations were analyzed, and content or production of DHA and TFA were determined.

In the experimental set-up 2, *T. lutea* was cultivated in mixotrophic and heterotrophic conditions, and in autotrophic condition as a control. Mixotrophic and autotrophic condition were in a 1-L bioreactor (Additional file 1: Fig. S1) as described elsewhere (Miao and Wu 2006). Briefly, 600 mL f/2-Si medium and 60 mL pre-culture microalgal suspension were transferred to the bioreactor. A glass tube was inserted into the bottom of the bioreactor to supply mixed gas (4% CO_2_ in air) at the rate of 0.25 v/v/min. The gas was sterilized by filtering through a 0.20-μm PTFE gas filter diaphragm (Midisart-2000, SRP65, Sartorius, Germany). The light conditions were same as in the pre-culture conditions. The heterotrophic condition was in a 1-L Erlenmeyer flask with 600 mL of the medium and 60 mL of the pre-culture microalgal suspension and no aeration and illumination. For the mixotrophy and heterotrophy, pure glycerol (Sinopharm Chemical Reagent Co., Ltd, China) was added to the f/2-Si medium at 5 g/L as an organic carbon source. The temperature and pH of the three culture modes were the same as in the pre-culture.

### Analytical methods

#### Determination of biomass

Twenty milliliters of microalgae solution was centrifuged at 8000 rpm (7656 g) for 8 min (4 °C) for microalgae cells. The cells were then washed twice by 0.5 M ammonium formate to eliminate salt residues. Then microalgal cells were filtered through a cellulose acetate filter membrane (0.45 μm) and then the filter membrane containing the biomass was dried at 105 °C to constant weight. The biomass of the microalgae was calculated by Eq. (1).

1 $${\text{Biomss (g/L) = }}\frac{{{\text{m}}_{ 1} ( {\text{g)}} - {\text{m}}_{ 0} ( {\text{g)}}}}{\text{V (L)}}$$

In Eq. (1), m_1_ is the dry weight of the blank filter membrane; m_2_ is the dry weight of loaded filter membrane, V is the volume of sample. And the growth rates were calculated by determining the slopes of the linear regression of the biomass over time.

#### Determination of nutrient concentrations

The culture broth was filtered through 0.45-μm filter paper and the filtrate was used to analyze the nutrient concentrations. The ${\text{NO}}_{3}^{-}{\text{-N}}$ (N) and ${\text{PO}}_{4}^{3-} {\text{-P}}$ (P) concentrations were determined by using a water quality auto-analyzer (Aquakem 200, Thermo Fisher Scientific, Finland) according to standard methods (Greenberg Arnold and Clesceri Lenore 1992). The glucose and glycerol concentrations in the culture media were determined by high-performance liquid chromatography system (HPLC, Agilent1200), equipped with refractive index detector (RID) and Aminex HPX-87H column (7.8 × 300 mm) at 45 °C. Mobile phase was 4 mM H_2_SO_4_ with a flow rate 0.5 mL/min. All samples were diluted to be < 1 g/L substrate in concentration, and the injection volume was 20 μL. The parameters were set according to Babuskin et al. (2014). For sodium acetate concentration, filtrate of the samples were acidified by a formic acid with 3% (v/v) and diluted to be < 1 g/L, then was analyzed with a gas chromatography system (GC, Agilent 7890) equipped with a flame ionization detector (FID) and a DB-FFAP fused-silica capillary column (30 m × 0.25 mm × 0.25 μm). The GC parameters were as follows: injection volume 1 μL; split ratio 1:10; air 400 mL/min, H_2_ 40 mL/min, gas carrier (N_2_) 45 mL/min; injector temperature 250 °C; detector temperature 300 °C; oven temperature started at 70 °C for 3 min and was raised to 180 °C at a rate of 10 °C/min, and was then held for 4.5 min. The parameters were based to the methods by Chen et al. (2016). The assimilation rates of the ${\text{NO}}_{3}^{-}{\text{-N}}$ ${\text{PO}}_{4}^{3-} {\text{-P}}$, glucose, glycerol and acetate were calculated by reduction divided by the original concentration.

#### Determination of DHA and TFAs

The FAs including DHA were determined as fatty acid methyl esters after direct transesterification using a method described by Rodriguez-Ruiz et al. (1998). Lyophilized microalgae powder (about 20 mg) was collected for transesterification. The detailed steps and operating conditions ware similar to the method described by Chu et al. (2013). After transesterification, 2 mL methyl benzoate (about 0.44 mg/mL in hexane) was added as an internal standard. Then the samples were analyzed for the FA content by gas chromatography system (GC, Agilent 6890, CA). The GC system equipped with a flame ionization detector (FID) and a Supelco DB-FFAP capillary column (30 m × 0.25 mm × 0.25 μm). Chromatographic parameters were as follows: injection volume 1 μL; split ratio 1:10; air 450 mL/min, gas carrier (N_2_) 45 mL/min, H_2_ 40 mL/min; injector temperature 250 °C; detector temperature 300 °C; oven temperature started from 140 °C for 2 min, then increased to 240 °C with a rate of 10 °C/min, then held at 240 °C for 2 min. The parameters were based to the methods described by Chu et al. (2013). The FAs besides the DHA were identified by the peak retention times compared with that of standard FAs and concentration of FAs was determined by calibration curves which were based on the peak area of standard FAs comparing with the peak area of internal standard (methyl benzoate). Then dry weight of FAs (besides the DHA) was determined by this concentration and extraction volume. Dry weight of TFAs was the sum of each FA dry weight in this study.

The content and production of DHA and TFA were calculated by Eqs. (2–5).

2 $${\text{DHA or TFA content (mg/g)}} = \frac{{{\text{DHA or TFA dry weight}}\text{(mg)}}}{{{\text{ dry weight of biomass}}\text{(g)}}}$$

3 $$\begin{aligned} & {\text{DHA or TFA production (mg/L) = }} \\ & {\text{ DHA or TFA content (mg/g)}} \times {\text{Biomass (g/L)}} \\ \end{aligned}$$

4 $$\begin{aligned} &{\text{DHA or FA proportion (\% ) =}} \\ & \frac{\text{DHA or FA content (mg/g)}}{{{\text{ TFA content (mg/g}})}} \\ \end{aligned}$$

5 $$\begin{aligned} & {\text{DHA or TFA productivity (\% ) }} \\ & = \frac{{\left[ {\text{DHA or TFA production (mg/L)}} \right]_{\text{t1}} - \left[ {\text{DHA or TFA production (mg/L)}} \right]_{\text{t0}} }}{{{\text{ t1}} - {\text{t0 (d)}}}} \\ \end{aligned}$$

The DHA or TFA yield (% COD) was calculated based on COD coefficient (g O_2_/g organic compound) which was the amount of O_2_ needed to oxide the organic compounds. COD coefficient of glycerol and DHA or FAs (CxHyOz) can be calculated by Eq. (6).

6 $${\text{COD coefficient (CxHyOz)}} = \frac{{{\text{ 32x}} + 8 {\text{y}} - 1 6 {\text{z}}}}{{{\text{ 12x}} + {\text{y}} + 1 6 {\text{z}}}}$$

Then DHA or FA yield (% COD) can be determined by Eq. (7), and TFA yield (% COD) was the sum of all FA yield (% COD) in this study.

7 $$\begin {aligned} &{\text{DHA or FA yield (\% COD)}} \\ &= \frac{{{\text{ COD coefficient}}_{\text{DHA or FA}} \times {\text{DHA or FA production (g)}}}}{{{\text{ COD coefficient}}_{\text{Glycerol}} \times {\text{ Glycerol assumption (g)}}}}\end {aligned}$$

### Statistical analysis

The data analysis was performed using Microsoft Excel. The data were analyzed using IBM SPSS (Statistical Product and Service Solutions) by one-way analysis of variance (ANOVA) combined with a least squares difference (LSD) analysis and the effective value was greater than the 95% confidence interval, namely a *p* value that was less than or equal to 0.05.

## Results

### Microalgal growth on three organic carbon substrates

The microalgal growth rates and organic carbon substrate assimilation of the three carbon source groups are shown in Table 1. The microalgal growth rate was slightly higher for the glycerol group (20.0 mg/L/day) than the control group (18.3 mg/L/day). The N and P assimilation rate was significantly higher for the glycerol group than the control group (*p* < 0.05) and the glycerol assimilation rate was about 2%. In contrast, the microalgal growth rates for the glucose and acetate groups were 5.8 and 1.9 mg/L/day, respectively and were much lower than the control group. The N and P assimilation rate were lower for the glucose and acetate groups than the control group. The assimilation rates of glucose and acetate were almost zero. The DHA and TFA production were lower for the glucose and acetate groups than the glycerol and control groups (Additional file 1: Fig. S2). The results clearly demonstrated that the glycerol was an appropriate organic carbon source for *T. lutea* growth. Therefore, mixotrophic and heterotrophic culture for *T. lutea* using glycerol as organic source were investigated as follows.

**Table 1:** Microalgal growth rate and nutrient assimilation during 10-day cultivation in mixotrophy using three carbon substrates

Carbon substrates	Growth rate (mg/L/day)	N assimilation rate (%)	P assimilation rate (%)	Glucose assimilation rate (%)	Glycerol assimilation rate (%)	Acetate assimilation rate (%)
Glucose	5.8 ± 2.1^B^	20.6 ± 2.7^C^	35.8 ± 7.0^B^	0	–	–
Glycerol	20.0 ± 0.8^A^	31.4 ± 0.3^A^	61.5 ± 9.6^A^	–	2.0 ± 0.5	–
Acetate	1.9 ± 0.6^C^	0.0^D^	5.9 ± 2.3^C^	–	–	0
No carbon^a^	18.3 ± 0.0^A^	24.7 ± 0.0^B^	42.3 ± 0.0^B^	–		–

The values from three biological replicates are expressed as mean ± one standard deviation. Statistical analysis was conducted using one-way ANOVA combined with LSD analysis at *p* < 0.05

^a^ No carbon means no carbon substrates in the medium, as a control group

^A, B, C, D^ Different capital letters denote a significant difference

### Microalgal growth in three cultivation modes

**Fig. 1:**
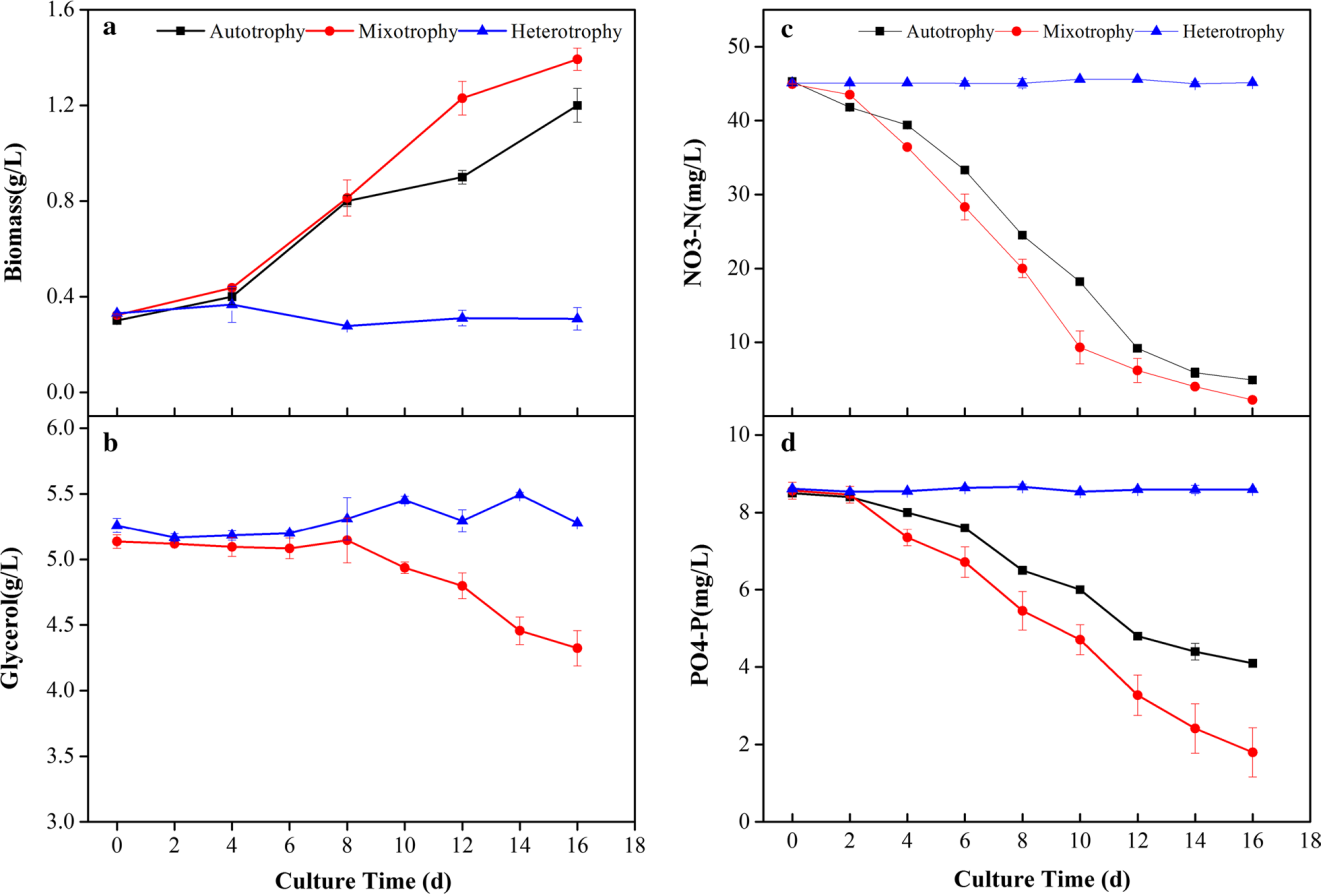
Biomass production and nutrient assimilation of *T. lutea* in three modes of culture. **a** Biomass, **b** glycerol, **c** ${\text{NO}}_{3}^{-} {\text{-N}}$, **d** ${\text{PO}}_{4}^{3-} {\text{-P}}$. The values from three biological replicates are expressed as mean ± one standard deviation

The microalgal biomass in the mixotrophic culture with the glycerol increases for the first 8 days and growth amount was almost on a par with autotrophic culture. However, after day 8, the biomass was higher for the mixotrophy than the autotrophy and reached 1.4 g/L on day 16 (Fig. 1a). However, the biomass in the heterotrophic culture almost remained unchanged for the 16 days (Fig. 1a). The cell morphology observations by a 100-times objective of the lens indicated differences in the cell sizes of the three culture modes (Additional file 1: Fig. S3). The consumption of nitrogen and phosphorus was similar for the mixotrophy and the autotrophy for the first 8 days (Fig. 1c, d). After day 8, the consumption of nitrogen and phosphorus was higher for the mixotrophic group than the autotrophic group, while no apparent changes were observed in the heterotrophic group. This trend corresponds to the growth of the biomass in the three culture modes in Fig. 1a. The consumption of glycerol in the mixotrophy remained almost unchanged from 0 to 8 days but began to fall after day 8 and exhibited a 0.8 g/L decrease at 16 days (Fig. 1b). The trends corresponded to the biomass growth and nitrogen and phosphorus assimilation in the mixotrophy. However, the glycerol concentration almost remained unchanged in the heterotrophy during the 16 days (Fig. 1b).

### DHA content and production

**Fig. 2:**
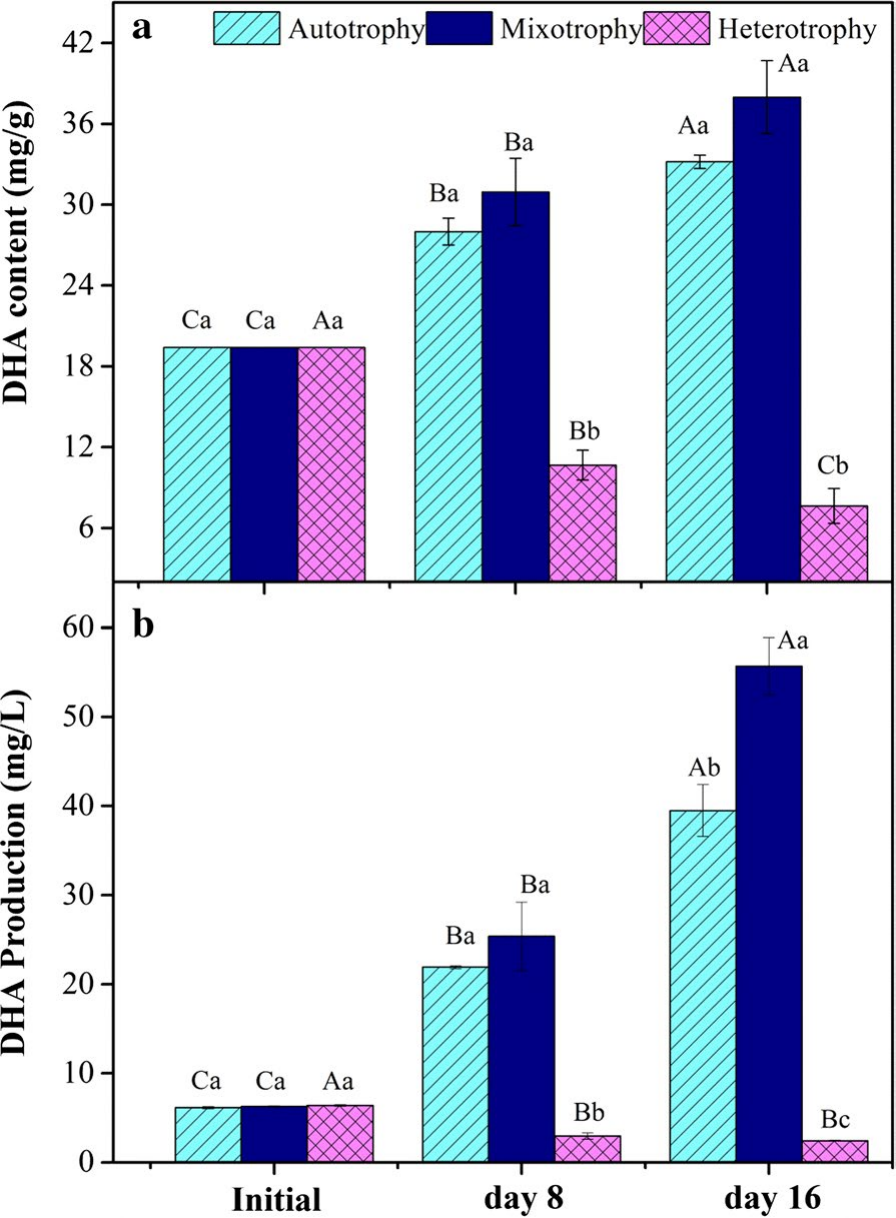
Content in dry weight and production in medium of DHA from *T. lutea* in three culture modes. **a** Content, **b** production. The values from three biological replicates are expressed as mean ± one standard deviation. Various capital letters denote significant differences among the values of the same condition on different days; different lowercase letters denote significant differences among the values of three culture modes at the same time

The DHA content was not significantly higher in the mixotrophy than in the autotrophy (*p* > 0.05), while the heterotrophic group had a significantly lower DHA content than the autotrophy (*p* < 0.05) on the 8th day (Fig. 2a). The DHA content in the mixotrophy continued to increase from day 8 to day 16 and the mixotrophy values (37.98 mg/g) were slightly higher than the values for the autotrophy (33.18 mg/g) at the end of 16-day cultivation. But the DHA content in the heterotrophy declined slightly from 8 to 16 days. The DHA production exhibited a similar trend as the DHA content but it was significantly higher for the mixotrophy than for the autotrophic group on day 16 (*p* < 0.05) as shown in Fig. 2b.

The DHA productivities and yields (% COD) of the three culture modes were calculated during the 16-day cultivation and are shown in Table 2. During 8 days, the DHA productivity was not significantly higher for the mixotrophy than the autotrophy (*p* > 0.05) and was negative for the heterotrophy as shown in Table 2. In contrast, the DHA productivity from day 8 to 16 was nearly twofold higher for the mixotrophy than the autotrophy. During the same period, the DHA productivity for the heterotrophy was still negative. In addition, the DHA yields (% COD) in the mixotrophy as calculated by Eq. 5 were very low (2.63%) (Table 2).

**Table 2:** DHA and TFA productivity from *T. lutea* in three culture modes during the 16-day cultivation

Culture mode	DHA productivity (mg/L/day)		DHA yield (% COD)	TFA productivity (mg/L/day)		TFA yield (% COD)
	0–8 days	8–16 days	16 days	0–8 days	8–16 days	16 days
Autotrophy	1.97 ± 0.01^A^	2.20 ± 0.17^B^	–	16.04 ± 0.39^A^	17.28 ± 0.04^B^	–
Mixotrophy	2.38 ± 0.07^A^	3.79 ± 0.19^A^	2.63 ± 011	18.60 ± 0.67^A^	26.75 ± 0.55^A^	13.10 ± 1.90
Heterotrophy	− 0.43 ± 0.03^B^	− 0.07 ± 0.00^C^	–	− 2.80 ± 0.03^B^	0.055 ± 0.01^C^	–

The values from three biological replicates are expressed as mean ± one standard deviation. Statistical analysis was conducted using one-way ANOVA combined with LSD analysis at *p* < 0.05

^A, B, C, D^ Different capital letters denote a significant difference

### TFA production and composition

**Fig. 3:**
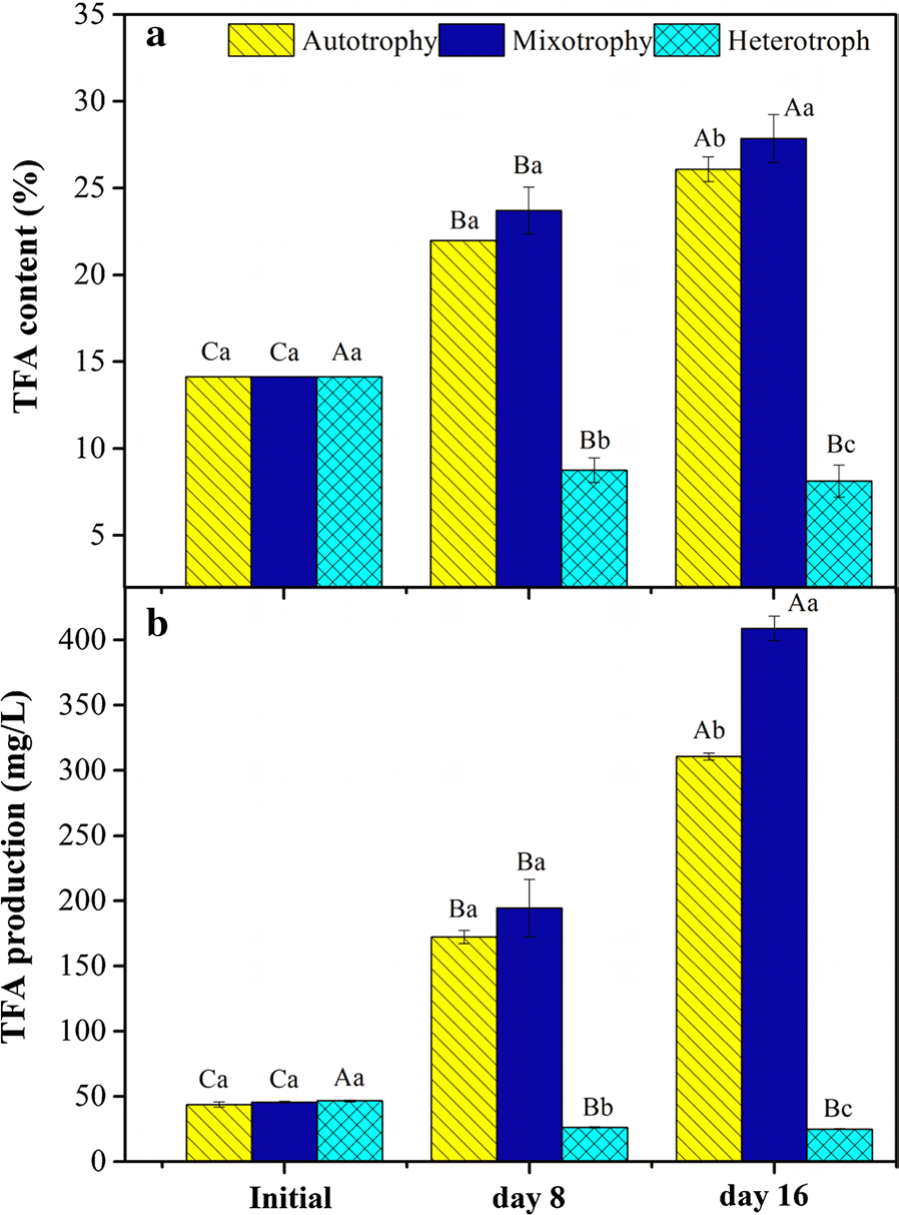
TFA content in dry weight and production in medium by *T. lutea* in three culture modes. **a** Content, **b** production. The values from three biological replicates are expressed as mean ± one standard deviation. Various capital letters denote significant differences among the values of the same condition on different days; different lowercase letters denote significant differences among the values of three culture modes at the same time

**Fig. 4:**
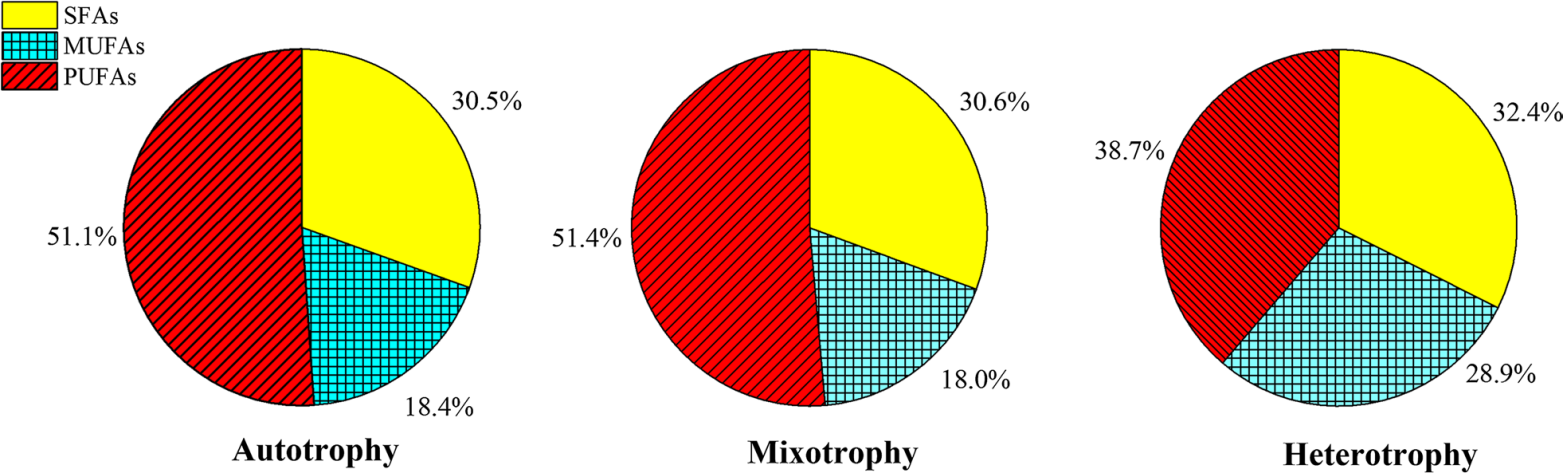
Three categories of FA proportions in TFAs under three culture modes at the end of 16-day cultivation

The TFA production and composition are shown in Figs. 3, 4 and Table 3. The TFA content in the mixotrophy increased significantly from 14.1% on day 0 to 23.7% at day 8. A similar TFA content was observed for the autotrophy during the same period (*p* > 0.05). On the other hand, the TFA content in the heterotrophy decreased from 14.1 to 8.7% and was significantly lower than the TFA content observed in the autotrophy (*p* < 0.05) during initial 8-day cultivation (Fig. 3a). From day 8 to 16, the TFA content in the mixotrophy increased and was noticeably higher than the value obtained in the autotrophy (*p* < 0.05). The TFA content in the heterotrophy changed slightly from day 8 to 16 (Fig. 3a). The TFA production of the mixotrophy was not obviously higher than the autotrophy (*p* > 0.05) at day 8. And it was significantly higher than the autotrophy (*p* < 0.05) at the end of the 16-day cultivation (Fig. 3b). On the contrast, the TFA production of the heterotrophy was much lower than the autotrophy (*p* < 0.05) at day 8 and day 16. During the first 8 days, the productivity was slightly higher in the mixotrophy (18.60 mg/L/day) than the autotrophy (16.04 mg/L/day) and was negative in the heterotrophy (Table 2). The TFA productivity was 54% higher in the mixotrophy than the autotrophy and was almost zero in the heterotrophy during the last 8 days. The TFA yield (% COD) in the mixotrophy was also not high (13.10%) from Table 2.

**Table 3:** FA composition, content and proportion of *T. lutea* after the 16-day cultivation under three culture modes

FA	Autotrophy		Mixotrophy		Heterotrophy	
	Content (mg/g)	Proportion (%)	Content (mg/g)	Proportion (%)	Content (mg/g)	Proportion (%)
SFAs						
C13:0	1.89 ± 0.00	0.72 ± 0.00	1.79 ± 0.32	0.64 ± 0.11	0.65 ± 0.01	0.80 ± 0.01
C14:0	46.17 ± 0.3	17.70 ± 0.11	49.46 ± 2.00	17.75 ± 0.72	11.49 ± 0.07	14.13 ± 0.09
C15:0	0.58 ± 0.06	0.22 ± 0.02	1.09 ± 0.11	0.39 ± 0.04	ND	ND
C16:0	26.53 ± 0.97	10.17 ± 0.37	28.86 ± 3.54	10.36 ± 1.27	10.93 ± 0.12	13.44 ± 0.15
C17:0	2.41 ± 0.04	0.92 ± 0.02	2.22 ± 0.06	0.80 ± 0.09	ND	ND
C18:0	1.39 ± 0.14	0.53 ± 0.05	0.83 ± 0.08	0.30 ± 0.14	0.79 ± 0.02	0.97 ± 0.02
C20:0	0.64 ± 0.04	0.24 ± 0.02	1.00 ± 0.11	0.36 ± 0.08	2.50 ± 0.03	3.07 ± 0.03
Sum	79.6 ± 1.56	30.50 ± 0.59	85.25 ± 1.82	30.60 ± 0.45	26.35 ± 0.24	32.41 ± 0.30
MUFAs						
C16:1	12.66 ± 0.15	4.85 ± 0.06	13.31 ± 2.2	4.78 ± 0.79	9.14 ± 0.13	11.24 ± 0.16
C17:1	0.82 ± 0.01	0.31 ± 0.00	0.79 ± 0.14	0.28 ± 0.00	2.59 ± 0.05	3.18 ± 0.06
C18:1	33.31 ± 0.48	12.77 ± 0.18	34.92 ± 2.77	12.54 ± 1.51	10.86 ± 0.66	13.36 ± 0.81
C20:1	1.16 ± 0.01	0.44 ± 0.01	1.10 ± 0.03	0.40 ± 0.01	0.92 ± 0.07	1.14 ± 0.09
Sum	47.94 ± 0.65	18.37 ± 0.25	50.13 ± 12.14	18.00 ± 0.36	23.51 ± 0.91	28.92 ± 0.12
PUFAs						
C18:2	29.07 ± 0.73	11.15 ± 0.28	34.21 ± 10.49	12.28 ± 0.77	7.4 ± 0.11	9.10 ± 0.14
C18:3	27.09 ± 0.01	10.39 ± 0.01	22.49 ± 1.74	8.07 ± 0.62	3.95 ± 0.00	4.86 ± 0.00
C18:4	33.59 ± 0.33	12.88 ± 0.13	37.02 ± 1.61	13.29 ± 0.58	11.79 ± 0.01	14.5 ± 0.01
C20:4	0.94 ± 0.04	0.36 ± 0.02	1.19 ± 0.22	0.43 ± 0.08	ND	ND
C20:5	3.87 ± 0.1	1.49 ± 0.04	3.59 ± 0.55	1.29 ± 0.20	ND	ND
C22:5	5.51 ± 0.09	2.11 ± 0.04	6.35 ± 0.07	2.28 ± 0.02	0.69 ± 0.97	0.85 ± 1.20
C22:6	33.18 ± 0.45	12.72 ± 0.17	38.37 ± 0.37	13.77 ± 0.13	7.62 ± 0.30	9.37 ± 0.37
Sum	133.26 ± 1.76	51.10 ± 0.69	143.22 ± 15.05	51.40 ± 0.50	31.44 ± 1.39	38.68 ± 0.72

The values from three biological replicates are expressed as mean ± one standard deviation

*SFAs* saturated fatty acids, *MUFAs* monounsaturated fatty acids, *PUFAs* polyunsaturated fatty acids, *ND* not detected

At the end of the 16-day cultivation, the main FA content for dry weight and its proportion in TFAs of the three culture modes are shown in Table 3. The FAs were divided into saturated FAs (SFAs), monounsaturated FAs (MUFAs), and polyunsaturated FAs (PUFAs). The proportions of these three kinds of FAs were shown in Fig. 4. The proportions of the three kinds of FAs were comparable in the mixotrophy and the autotrophy. The PUFAs had the highest proportion with 51.1 and 51.4% for the autotrophy and the mixotrophy, respectively, followed by the SFAs with 30.5 and 30.6% for the autotrophy and the mixotrophy, respectively and the MUFAs with 18.0 and 18.4% in the mixotrophy and the autotrophy, respectively (Fig. 4). The SFAs was slightly higher and the MUFAs are 60% higher in the heterotrophy, while the PUFAs was significantly lower (34.3%) than the autotrophy. However the ranking of the three kinds of FAs was the same for the three culture modes.

## Discussion

### Organic carbon source usage

Glucose or acetate is a widely used carbon source for DHA or TFA production by certain marine microalgae or microalgae-like microorganisms (Chi et al. 2009; De Swaaf et al. 2003) and for biodiesel production by certain microalgae (Shen et al. 2015a, b) in heterotrophic or mixotrophic conditions. However the two carbon sources were unable to maintain the mixotrophic growth of *T. lutea* as shown in Table 1. Traditionally, *T. lutea* is an autotrophic microalga rather than a heterotrophic or mixotrophic microalga with regard to the organic carbon sources. However, glycerol as a carbon source increased the biomass of *T. lutea* during 10-day cultivation. The utilization of different carbon sources such as glucose, acetate, and glycerol either by mixotrophy or heterotrophy is deemed to be species dependent and depends on the presence of specific transporters or permeases (Gupta et al. 2016). Therefore, it could be deduced that *T. lutea* has glycerol transporters that exclusively transport glycerol rather than glucose or acetate into the microalgal cells, where it is further broken down in the mitochondria by oxidative phosphorylation to generate adenosine triphosphate (ATP) (Perez-Garcia et al. 2011) and promote growth. Moreover, *T. lutea* used glycerol only in mixotrophic conditions and no use of glycerol occurred in heterotrophic conditions (Fig. 1b). This indicates that the glycerol transporters in *T. lutea* only function under light illumination, which furthermore suggests that photosynthesis may stimulate the function of glycerol transporters; however, the mechanism is still not very clear.

The biomass growth and the consumption of nitrogen, phosphorus, and glycerol (Fig. 1) indicated that *T. lutea* using glycerol has a period of adaptation under mixotrophic growth. In other words, the mixotrophic growth of *T. lutea* using glycerol was a two-stage growth. The first stage (day 0 to day 8) was an autotrophic growth and the second stage (day 8 to day 16) was a mixotrophic growth. Alkhamis and Qin (2013) also reported a 2-day adaptation period for *Isochrysis galbana* (*T. lutea affinis* species) and after 2 days, the glycerol was being used. Some other microalgae exhibited similar behavior such as *P. tricornutum* studied by Cerón Garcí et al. (2000) and *Haematococcus* sp., *Chlorella* sp., and *Scenedesmus* sp. reported by Andruleviciute et al. (2014). Therefore it may be a common phenomenon that microalgae using organic substrates have an adaptation period when an autotrophic cultivation is converted into mixotrophic conditions. Liu et al. (2009) found that organic carbon sources decreased the net photosynthetic O_2_ evolution of *P. tricornutum* but increased the respiration rate, consequently, a long adaption period was required, which maybe explain the adaption behavior of these microalgae.

### DHA production under three cultivation modes

Corresponding with the two-stage growth, two stages in the DHA production and TFA accumulation in the mixotrophic growth by *T. lutea* was also observed. Figures 2 and 3 indicate that the DHA and TFA production are not noticeably higher in the mixotrophy than the autotrophy during 0–8 days but are clearly higher in the mixotrophy during 8–16 days. The trend of DHA and TFA productivity (Table 2) in the mixotrophy is similar to that of DHA and TFA production.

In previous studies, mostly autotrophic culture have been used to produce DHA or TFA by *T. lutea* while the mixotrophic and heterotrophic cultures were seldom used as shown in Table 4. The DHA content in the autotrophy in this study (37.98 mg/g) is clearly higher than in other studies of autotrophy (15–22 mg/g). The most likely reason was that in this study, air with 4% CO_2_ was used while air with no or low CO_2_ (1%) was used in previous studies. Shene et al. (2016) also indicated that the eicosapentaenoic acid (EPA) content in the biomass and the production in the medium increased for high-level CO_2_ compared to no or low CO_2_ during the study of *Nannochloropsis oculata*. Therefore higher DHA production under autotrophic culture in this study might be attributed to appropriate CO_2_ ratio in air.

**Table 4:** DHA and TFA production from *T. lutea* in the literature and in this study

Culture mode	Aeration	DHA/TFA (%)	DHA content (mg/g)	DHA production (mg/L)	TFA content (% DW) and production (mg/L)	Refs.
Autotrophy	Air	14%	21 mg/g	NR	15%	Tzovenis et al. (1997)
Autotrophy	NR	12.2%	18.1 mg/g	5.8 mg/L	14.74% and 47.5 mg/L	Tzovenis et al. (2003)
Autotrophy	No	8–13%	15–17 mg/g	NR	13%	Mulders et al. (2013)
Autotrophy	Air with 1% CO_2_	8.5%	NR	16.1 mg/L	224 mg/L	Nalder et al. (2015)
Autotrophy	Air with 1% CO_2_	8.2%	NR	41 mg/L	500 mg/L	Rasdi and Qin (2015)
Autotrophy Mixotrophy	Air	Autotrophy 10.1% Mixotrophy 8.6%	Autotrophy 22.22 mg/g Mixotrophy 16.59 mg/g	Autotrophy 8.36 mg/L Mixotrophy 13.94 mg/L	Autotrophy 22% and 83.6 mg/L Mixotrophy 19.3% and 162.1 mg/L	Alkhamis and Qin (2016)
Atotrophy Mixotrophy Heterotrophy	Air with 4% CO_2_	Autotrophy 12.7% Mixotrophy 13.8% Heterotrophy 9.4%	Autotrophy 33.18 mg/g Mixotrophy 37.98 mg/g Heterotrophy 7.64 mg/g	Autotrophy 39.50 mg/L Mixotrophy 55.69 mg/L Heterotrophy 2.43 mg/L	Autotrophy 26.1% and 310.7 mg/L Mixotrophy 28.0% and 408.5 mg/L Heterotrophy 8.1% and 24.7 mg/L	This study

DW means cell dry weight

*NR* no record

Alkhamis and Qin (2016) obtained 16.3 mg/g DHA content and 13.9 mg/L DHA production in the mixotrophy and these values were much lower than the values obtained in this study (37.98 mg/g and 55.69 mg/L) in the mixotrophy (Table 4). The greatest difference between the two studies was the glycerol/nitrogen ratio or carbon/nitrogen (C/N) ratio [92.5 in this study vs. 18.4 in study of Alkhamis and Qin (2016)]. Singhasuwan et al. (2015) found a high C/N ratio greatly promoted the TFA content of *Chlorella* sp. under the heterotrophy. Therefore, it seems a very plausible hypothesis that the increase in the glycerol/nitrogen ratio facilitated the lipid synthesis and furthermore improved the DHA production in *T. lutea* under the mixotrophy; however, the mechanism has to be confirmed.

Rasdi and Qin (2015) found the N:P ratio influenced the DHA and TFA production from *T. lutea*. DHA proportion reached maximum in moderate N:P levels (10:1–30:1), while the lipid (TFAs) content was maximum in the highest N:P level (120:1) in their work. In this study, N:P was the moderate level (12.5) for all the three culture modes (autotrophy, mixotrophy and heterotrophy), so it might bring the high DHA and TFA contents for these three culture modes. N and P were assimilated faster under mixotrophic culture than autotrophic culture from day 4 to day 16 (Fig. 1), while they were assimilated little in heterotrophic culture. As a result, biomass of mixotrophic culture increased more than autotrophic and heterotrophic cultures during the same period. Therefore DHA and TFA productions of mixotrophic culture were higher than those of autotrophic culture and heterotrophic culture, due to the higher content of DHA and TFA and biomass concentration in the mixotrophy.

**Fig. 5:**
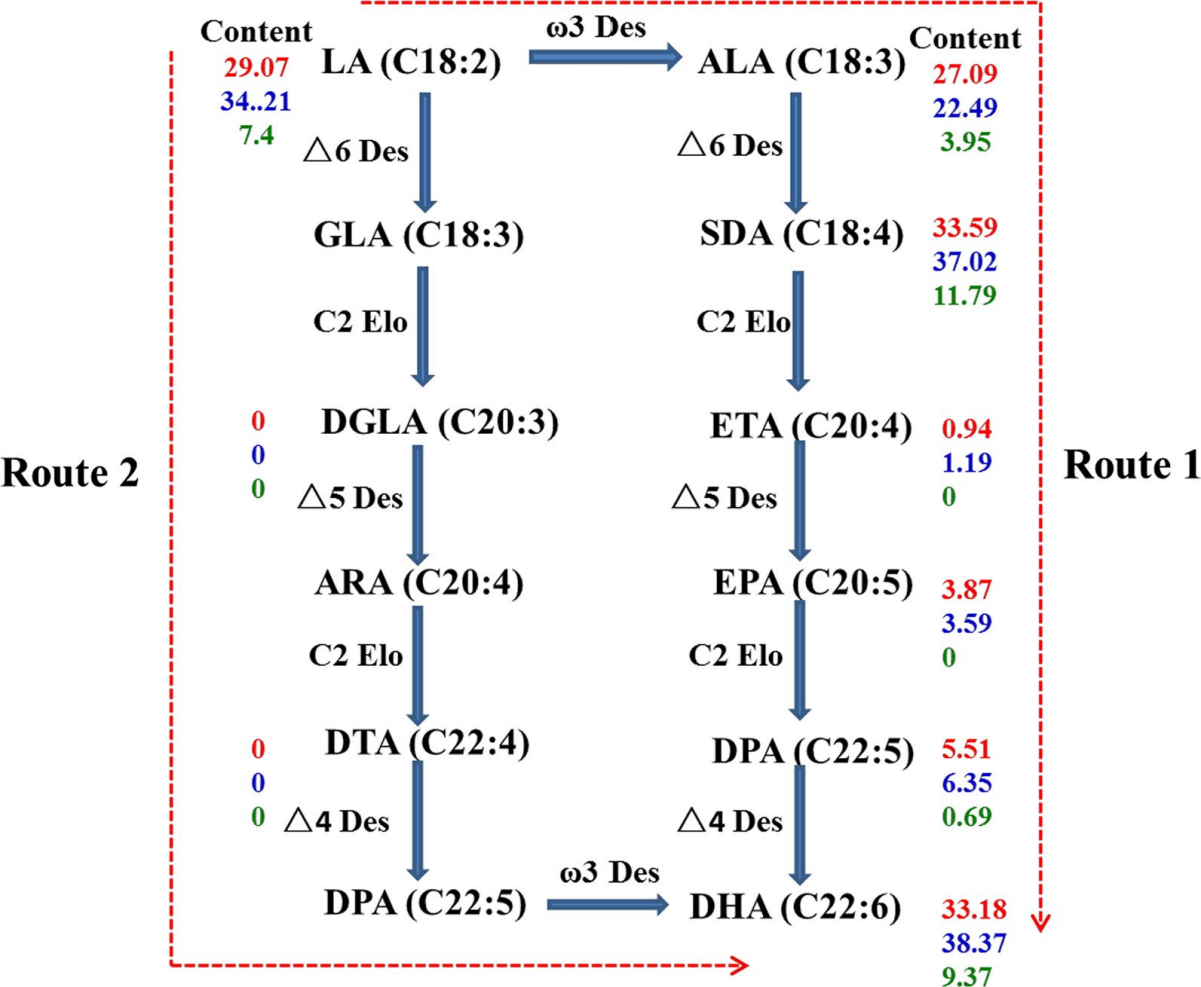
The conventional pathway of the DHA under three culture modes. These content values are dry weight contents of the long chain polyunsaturated fatty acids (LC-PUFAs) from Table 3. Red numbers (on the top) in content part denote the content in the autotrophy, blue numbers (in the medium) denote the content in the mixotrophy, and green numbers (at the bottom) denote the content in the heterotrophy. The abbreviations on the pathway are shown in the abbreviation list

The conventional pathway of DHA synthesis (Vaezi et al. 2013) has two routes from linoleic acid (LA, C18:2) to DHA by a consecutive series of desaturation and elongation steps as shown in Fig. 5. The di-homo γ-linolenic acid (DGLA, C20:3) and docosatetraenoic acid (DTA, C22:4) via Route 2 were not detected, however, all FAs via Route 1 were found in the three culture modes from Table 3. Therefore it was presumed that DHA accumulation under three culture modes was via Route 1. The contents of FAs on Route 1 were different under three culture modes. Some precursors of DHA via Route 1 such as LA, stearidonic acid (SDA, C18:4), eicosatetraenoic acid (ETA; 20:4), and docosapentaenoic acid (DPA; 22:5) in the mixotrophy were all higher than those in the autotrophy and the heterotrophy, thus, the DHA content of the mixotrophy was maximum at the end of cultivation.

The DHA and TFA production from the glycerol after the deduction of autotrophy contribution was not very high (16.19 and 97.8 mg/L, respectively) (Table 4). The DHA yield (% COD) was also very low (2.63%) and the TFA yield (% COD) was not high (13.10%) (Table 5) compared with other studies using other microalgae. The use of different microalgae might be responsible for this result. In view of this and the lag phase in the mixotrophy with the glycerol, *T. lutea* might be not an optimal strain for DHA production in mixotrophy. It may be possible to use metabolic engineering to shorten the lag phase of this microalga and achieve higher DHA yields (% COD). Shen et al. (2015a) reported earlier that *C. vulgaris* exhibited a higher FA yield (% COD = 87%) (Table 5) in heterotrophic cultivation with glucose but the longest chain FA was linolenic acid (C18:3). Therefore, glucose transporters or permeases (as described before) isolated from *C. vulgaris* may be implanted into *T. lutea* to improve the organic carbon source use and the conversion of the organic carbon to DHA. On the other hand, *C. vulgaris* lacks D6-desaturase or D6-elongase (Fig. 5) leading to an inability to synthesize EPA or DHA from C18:3 (Zhou et al. 2007). Therefore, metabolic engineering of *C. vulgaris* may be attempted using D6-desaturase or D6-elongase genes from *T. lutea* to increase the production of DHA.

**Table 5:** Comparison of DHA yield and TFA yield by % COD equivalent in the literature

Culture mode	Strain	Carbon substrate	DHA yield (% COD)	TFAs yield (% COD)	Refs.
Heterotrophy	*Chlorella vulgaris*	Glucose	NC	87	Shen et al. (2015a)
Heterotrophy	*Scenedesmus obliquus*	Sodium acetate	NC	6.6	Shen et al. (2015b)
Mixotrophy	*Isochrysis galbana*	Glycerol	NP	41.7^a^	Babuskin et al. (2014)
Mixotrophy	*Tisochrysis lutea*	Glycerol	2.63	13.10	This study

NC: The microalgae does not contain this substance

NP: Not presented in the literature

^a^ Deducing data according to the literature data

This study also found that the proportion of PUFAs (DHA is a PUFA) was not lower in the mixotrophy than the autotrophy (Fig. 4, Table 3), which was not in agreement with the results of the study by Alkhamis and Qin (2016). In addition, the proportion of the PUFAs decreased markedly, the MUFAs increased, and the SFAs slightly increased in the heterotrophy. These results clearly showed the mixotrophy by glycerol was not disadvantageous for PUFAs synthesis, while the heterotrophy by glycerol was not conducive to PUFAs synthesis compared with the autotrophy.

In summary, glucose or acetate as an organic carbon source was unable to maintain the mixotrophic growth of *T. lutea*, while glycerol as an organic carbon source promoted the biomass growth. Furthermore, *T. lutea* using glycerol had a period of adaptation in the mixotrophic growth. An increase in the glycerol/nitrogen ratio might be beneficial to promoting the lipid content and furthermore improved the DHA production in the mixotrophy. Although the mixotrophy enhanced the DHA content and production, the DHA production directly from the glycerol was not very high (16.19 mg/L) and the DHA yield (2.6% COD) and the TFA yield (13.1% COD) were also very low. Therefore, *T. lutea* might not be an optimal microalgal strain for DHA and TFA production from organic carbon.

## Additional file

**Additional file 1: Fig. S1.** The bioreactor for culture of *T. lutea* under autotrophic and mixotrophic condition. **Fig. S2.** Production and content of DHA and TFAs from *T. lutea* by different carbon substrates. Values shown are averages of averages of three samples ± standard deviation. (a): DHA content, production and proportion; (b): TFAs content and production. Acetate group found little content of DHA or TFAs, so not presented in the figure. **Fig. S3.** Cell morphology under three culture modes by100 times objective of the lens at the end of 16-days cultivation.

## Abbreviations

Δ6Des: Δ6-desaturases
Δ5Des: Δ5-desaturases
Δ4Des: Δ4-desaturases
ALA(C18:3): α-linolenic acid
ARA(C20:4): arachidonic acid
ATP: adenosine triphosphate
C2Elo: C2 elongation
COD: chemical oxygen demand
DGLA(C20:3): di-homo γ-linolenic acid
DHA: docosahexaenoic acid
DPA(C22:5): docosapentaenoic acid
DTA(C22:4): docosatetraenoic acid
DW: dry weight
EPA: eicosapentaenoic acid
ETA(C20:4): eicosatetraenoic acid
f/2-Si: f/2 medium with Na_2_SiO_3_ removed
FA: fatty acid
GLA(C18:3): γ-linolenic acid
LA(C18:2): linoleic acid
LC-PUFAs: long chain polyunsaturated fatty acids
MUFAs: monounsaturated fatty acids
N: nitrate nitrogen ($${\text{NO}}_{3}^{-} {\text{-N}}$$)
P: orthophosphate phosphorus ($${\text{PO}}_{4}^{3-} {\text{-P}}$$)
PUFAs: polyunsaturated fatty acids
SDA(C18:4): stearidonic acid
SFAs: saturated fatty acids
TFAs: total fatty acids
