# Supplementary material for: Effect of cultivation mode on the production of docosahexaenoic acid by Tisochrysis lutea
Source: AMB Express. 2018 Mar 30;8:50. doi: 10.1186/s13568-018-0580-9 (PMC5878155; doi:10.1186/s13568-018-0580-9)
Supplement: Supplementary file 1 — Additional file 1: Fig. S1. The bioreactor for culture of T. lutea under autotrophic and mixotrophic condition. Fig. S2. Production and content of DHA and TFAs from T. lutea by different carbon substrates. Values shown are averages of averages of three samples ± standard deviation. (a): DHA content, production and proportion; (b): TFAs content and production. Acetate group found little content of DHA or TFAs, so not presented in the figure. Fig. S3. Cell morphology under three culture modes by100 times objective of the lens at the end of 16-days cultivation. [file 13568_2018_580_MOESM1_ESM.pdf]

Supporting Information

For AMB Express

Effect of cultivation mode on the production of docosahexaenoic acid by *Tisochrysis lutea*

Hao Hu[1,2], Lin-Lin Ma[1,3], Jia-Yun Li[4], Xiao-Fei Shen[1,3], Hou-Feng Wang[1] , Raymond Jianxiong Zeng*[,1,3]

[1] CAS Key Laboratory of Urban Pollutant Conversion, Department of Chemistry, University of Science and Technology of China, Hefei 230026, P.R. China

[2]Anhui Water Conservancy College，Hefei 231603，P.R. China

[3] Advanced Laboratory for Environmental Research and Technology, USTC-CityU, Suzhou, 215123 P. R. China

[4] The First Affiliated Hospital of Anhui University of traditional Chinese Medicine

*: Corresponding author

Prof. Raymond Jianxiong Zeng

Fax: +86 551 63600203

Email: rzeng@ustc.edu.cn

Other authors' email:

Hao Hu (henchhoo@mail.ustc.edu.cn); Lin-Lin Ma (mll0713@mail.ustc.edu.cn); Xiao-Fen Shen (sxf0912@mail.ustc.edu.cn); Jia-Yun Li (lijiayun6666@163.com); Hou-Feng Wang (wanghf@mail.ustc.edu.cn)

There are 4 pages totally in supporting information including 3 figures.

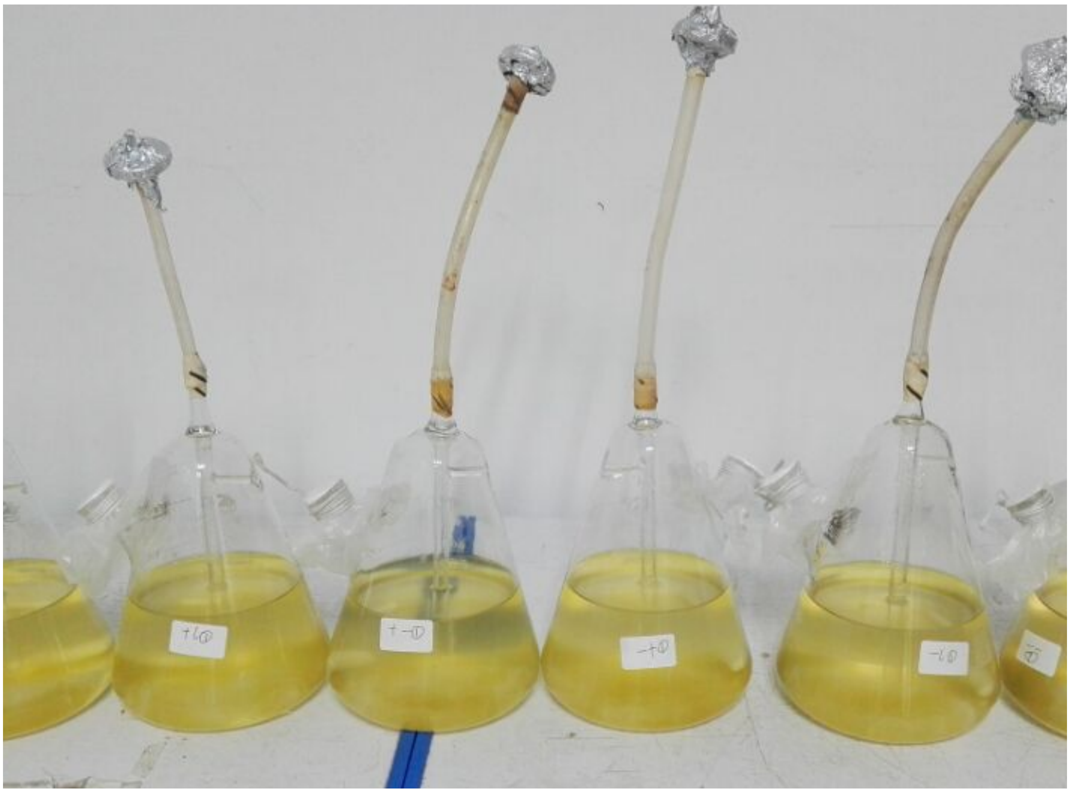

Fig. S1 The bioreactor for culture of *T. lutea* under autotrophic and mixotrophic condition

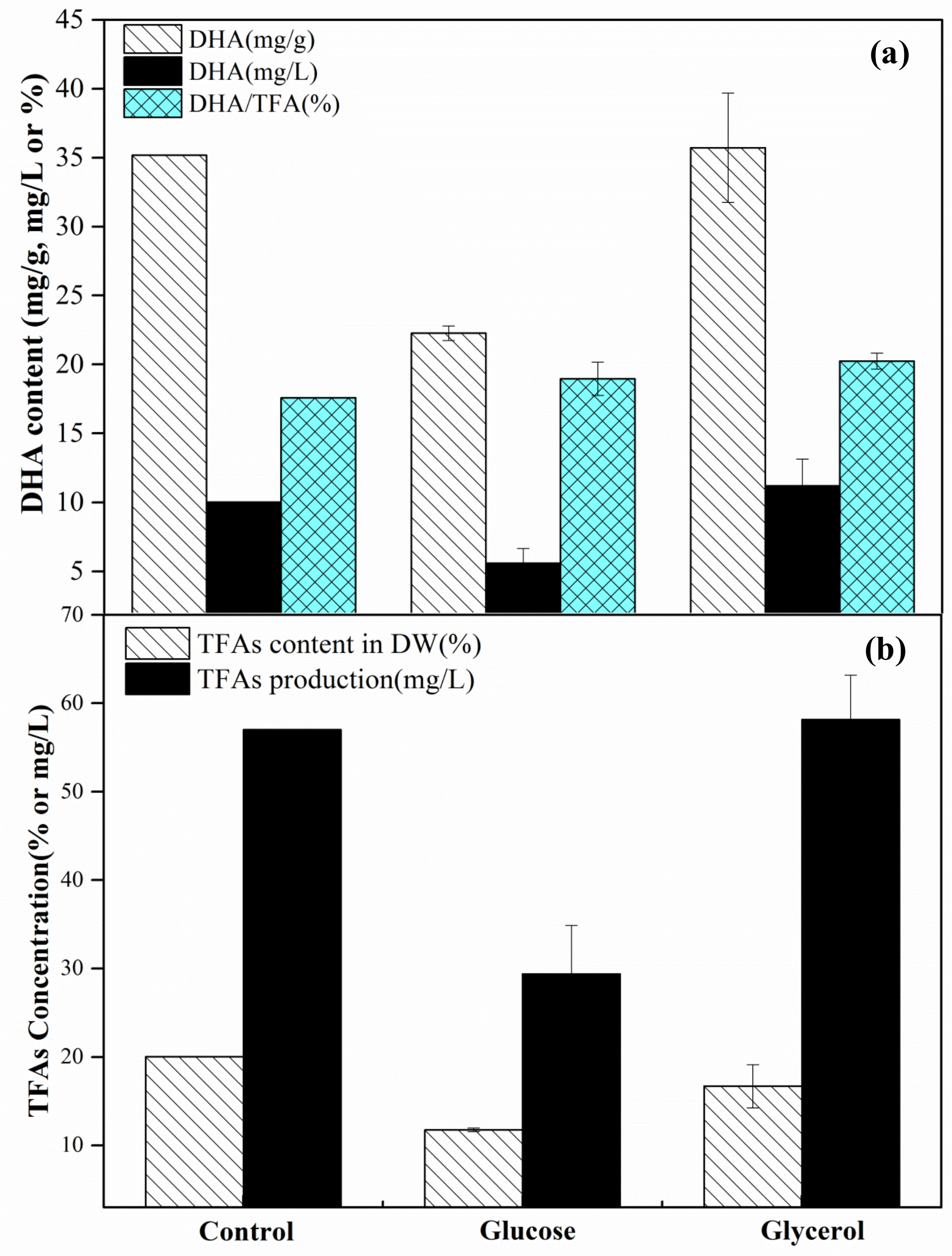

Fig. S2 Production and content of DHA and TFAs from *T. lutea* by different carbon substrates. Values shown are averages of averages of three samples ± standard deviation. (a): DHA content, production and proportion; (b): TFAs content and production. Acetate group found little content of DHA or TFAs, so not presented in the figure

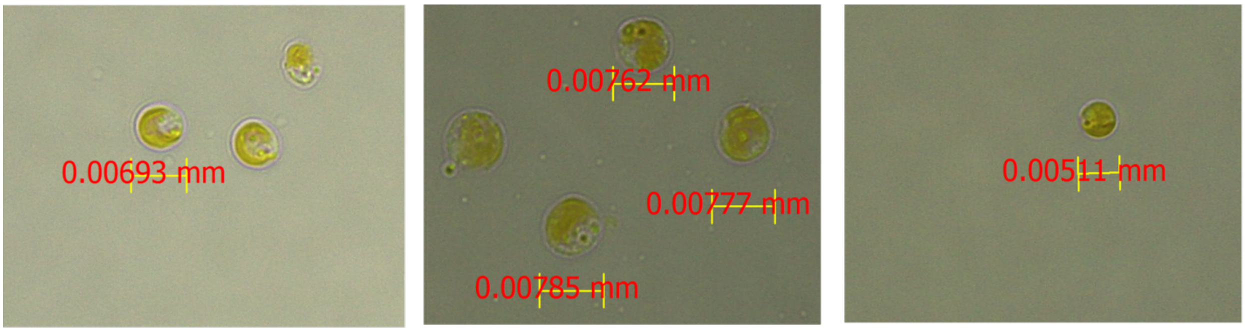

Autotrophy **Mixotrophy** **Heterotrophy**

Fig. S3 Cell morphology under three culture modes by100 times objective of the lens at the end of 16-days cultivation
